# Aortic Root Abscess Presenting As Chest Pain and Ischemic EKG Changes: Importance of Timely Diagnosis

**DOI:** 10.7759/cureus.38138

**Published:** 2023-04-26

**Authors:** Sardar Muhammad Alamzaib, Noor Ul Ann Rabbani, Zoya Sayyed, Kanaan Mansoor, Melissa D Lester

**Affiliations:** 1 Interventional Cardiology, Marshall University Joan C. Edwards School of Medicine, Huntington, USA; 2 Internal Medicine, Marshall University Joan C. Edwards School of Medicine, Huntington, USA; 3 Cardiology, Marshall University Joan C. Edwards School of Medicine, Huntington, USA

**Keywords:** intravenous drug user, transesophageal echo, aortic root abcess, perivalvular abcess, : acute coronary syndrome

## Abstract

The occurrence of perivalvular abscess, a purulent infection that affects the myocardium and endocardium of natural or artificial valve tissues, can result from either the spread of bacteria from a distant source via bacteremia, or from the expansion of an existing infectious cardiac focus, such as infective endocarditis (IE). The aortic abscess should be suspected in patients with infective endocarditis who fail to improve despite being on appropriate antibiotics. Sometimes aortic abscesses can present as PR interval lengthening or heart block due to the extension of infection. We present an atypical presentation of aortic root abscess with chest pain and ischemic EKG changes. A 45-year-old intravenous drug user presented with chest pain episodes with EKG showing ST depression in V2-V6 and ST elevation in avR. The coronary angiographic study showed no significant coronary artery disease, but the patient complained of chest pain. Transthoracic echo in the catheterization lab showed severe aortic regurgitation. The patient became hemodynamically unstable, worsened his respiratory status, and had to be intubated. He had a bedside transesophageal echo that revealed an aortic root abscess. The patient's condition continued deteriorating, and he passed away the same day. This case focuses on the timely diagnosis of aortic root abscess, and Transesophageal echocardiography (TEE) is the gold standard for diagnosing aortic root abscesses. This case also focuses on keeping perivalvular abscess among our differentials in a patient presenting with chest pain and abnormal EKG, especially in a high-risk population.

## Introduction

A perivalvular abscess is a severe infection that affects the heart valves and surrounding tissues. It can occur due to the spread of bacteria through the bloodstream, known as bacteremia, or as a complication of existing infections such as infective endocarditis (IE). A patient with IE may develop a perivalvular abscess in up to 30% of cases, with the aortic valve being the most frequently affected area [1]. Regarding clinical presentation, perivalvular abscess, especially aortic root abscess (ARA), may be characterized by a lengthening PR interval or the onset of heart block, depending on how far the infection has spread [2]. However, in rare cases, it may also manifest as chest pain with ischemic EKG changes, as was seen in our patient. This case describes a 45-year-old male who presented with chest pain and had no coronary artery disease on left heart catheterization but was found to have an aortic abscess on transesophageal echocardiography (TEE).

## Case presentation

**Figure 1:**
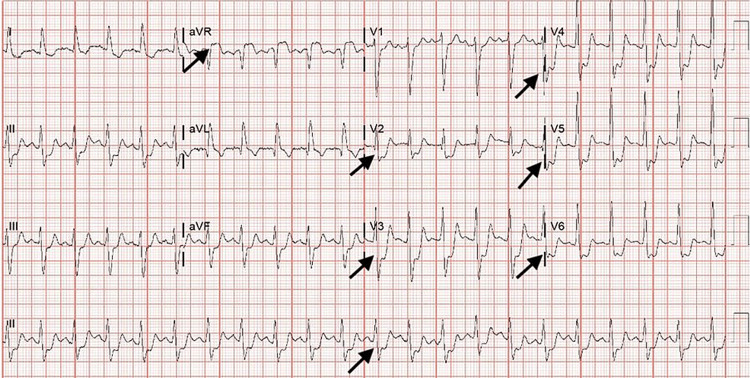
EKG showing severe ST depression in V2-V6 and ST elevation in aVR

**Figure 2:**
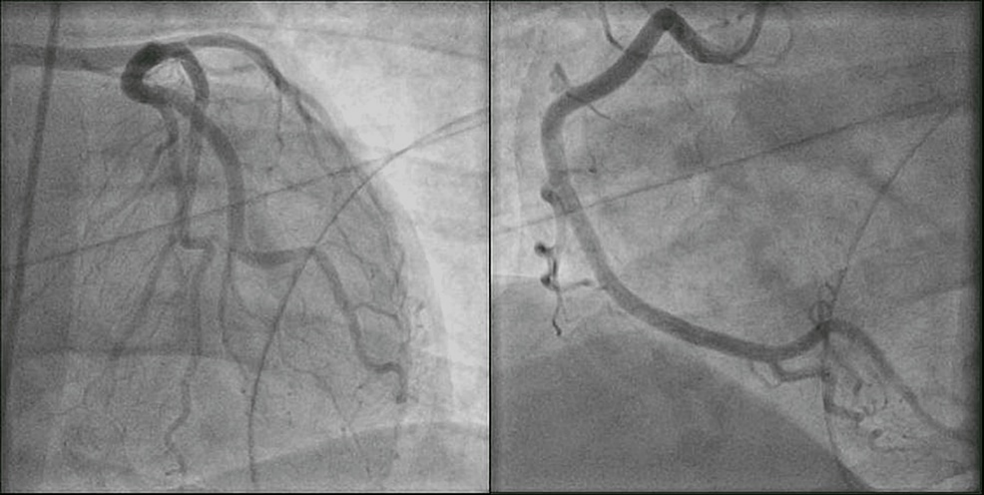
Normal Coronary Arteries on Left Heart Catheterization

**Figure 3:**
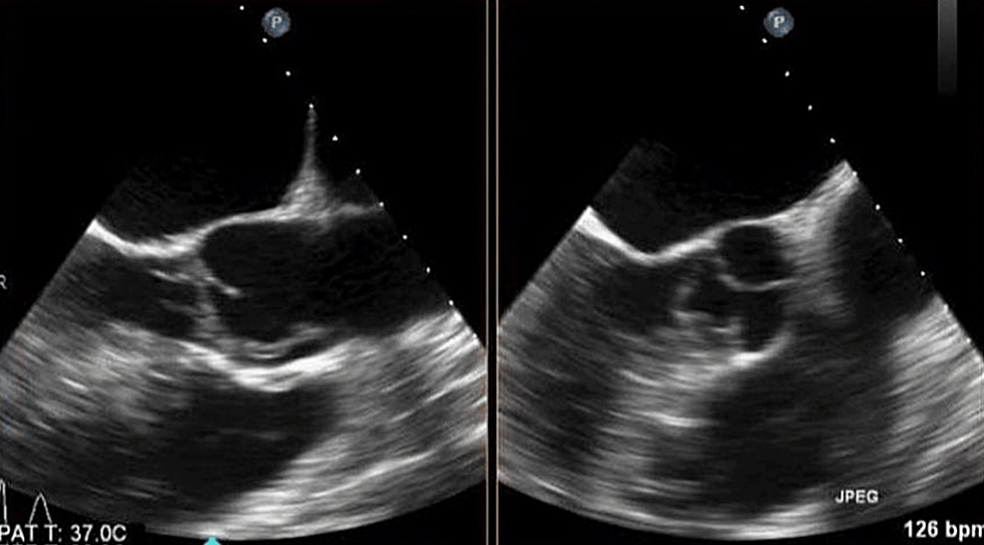
Transesophageal Echocardiogram showing Aortic Root Abscess

A 45-year-old male with a history pertinent to intravenous drug abuse was transferred from an outside hospital to evaluate chest pain, back pain, and EKG changes significantly for severe ST depression in V2-V6 and ST elevation in aVR (figure 1). His labs showed a WBC count of 42, Hemoglobin of 8.4, troponin 223, and sedimentation rate of 89. He was taken to the Cath lab emergently. A left heart catheterization revealed normal coronary arteries (figure 2), but the patient continued complaining of chest pain and back pain. Trans-thoracic Echo was done while the patient was in the Cath lab, showing severe aortic insufficiency. He was then taken directly for a chest CT. CT chest showed a linear defect within the ascending aorta extending from the aortic root, suspicious for type A dissection. The patient's respiratory status started declining, with his respiratory rate dropping to 50 and saturation dropping to 80s on 6L of oxygen. Anesthesiology was called, and the patient was intubated due to respiratory distress. TEE was performed after intubation. TEE revealed large, highly mobile vegetation on the aortic valve (figure 3), with evidence of aortic regurgitation. Cardiovascular and thoracic surgery was present at the bedside at the time of TEE. The patient's blood pressure started to drop soon after the TEE, and he had to be started on Norepinephrine and Vasopressin. The patient went into cardiac arrest soon after that. He was coded for about 45 minutes before the family decided to change the code status to do-not-resuscitate order (DNR), and he passed away. Blood culture later showed the growth of Streptococcus pyogenes.

## Discussion

ARA is a life-threatening complication of IE commonly caused by a single organism, such as Staphylococcus aureus or Escherichia coli, and is less commonly poly-microbial. Even with appropriate treatment, this complication has relatively high mortality ranging from 12.2% to 30% [3] and as high as 50% for prosthetic valve-associated ARA [4]. Risk factors associated with higher mortality included artificial valves, heart defects, a history of intravenous drug use, and delayed treatment. This highlights the importance of early diagnosis and high clinical suspicion of ARA.

ARA has a wide spectrum of clinical presentations, most classically consisting of a wide PR interval or complete heart block on electrocardiogram (ECG). Suppose a patient with infective endocarditis (IE) fails to show improvement within 72 hours of receiving suitable antibiotics. In that case, it is crucial to consider the possibility of aortic root abscess (ARA), particularly if the patient has a prosthetic valve, an ongoing increase in white blood cell (WBC) count, an elevation in other inflammatory markers like c-reactive protein (CRP), or if they develop skin abnormalities or embolic events despite receiving appropriate treatment. Our patient demonstrates the importance of high clinical suspicion as he presented with an uncommon presentation of ARA in the form of ischemic EKG changes. In the setting of IE, coronary ischemia is usually due to pre-existing coronary disease or can be due to embolism from vegetation. Another explanation is occlusion of the left anterior descending (LAD) and circumflex artery due to extrinsic compression by the aortic root abscess [5]. This compression can be caused by a subvalvular aneurysm or pseudo-aneurysm arising from a defect in the prosthesis suture line which is an occasional complication of aortic root surgery, especially in the setting of aortic root abscess [5,6]. Another possibility could be valve failure due to aortic root abscess in the setting of IE presenting with global sub-endocardial ischemia.

Diagnosing ARA is straightforward, as a trans-esophageal echocardiogram (TEE) is currently the gold standard [7]. TEE allows clear anatomical definition partially due to the greater proximity of the transducer to the heart, a higher signal-to-noise ratio, and the posterior part of the aortic ring lies in the far field in the acoustic shadow of an aortic prosthesis which is in the near field and is always imaged with high resolution. TEE has a sensitivity of 45%-89% with a specificity of 92-100% for diagnosing ARA [7]. Another workup that may be considered is ECG which can monitor for new AV blocks and has a positive predictive value of 88% for abscess formation; however, it has a low sensitivity of 45%. Dean et al. described a case in 1993 with an aortic root abscess causing unstable angina, which presented with ST segment depression in V5 and V6 on ECG, a rise in cardiac enzymes, and severe wall motion abnormalities on echocardiography suggesting a myocardial infarction [6].

The treatment for ARA is generally antibiotic coverage for stable patients. However, the duration of coverage is controversial, and then surgery as delayed treatment leads to a worse prognosis and increased mortality [7]. Although patients presenting with hemodynamic instability and refractory angina should undergo emergent surgery [5]. The best modality for pre-surgical planning is TEE because it can determine important surgical considerations, such as defining the extent of the abscess cavity and whether it has involved the sub-aortic curtain or upper inter-ventricular septum. The two most widely used techniques for the surgical treatment of ARA are aortic valve replacement (AVR) and aortic root replacement (ARR) [6].

## Conclusions

Aortic root abscess is common, but it is rare as chest pain with ischemic EKG changes can delay the diagnosis. Our patient initially did not present with typical symptoms of fevers or chills consistent with infective endocarditis. This case signifies keeping aortic root abscess as a differential for a patient presenting with ischemic EKG changes.
